# Distal tibial tuberculous osteitis in a child: a case report

**DOI:** 10.1093/jscr/rjag666

**Published:** 2026-08-31

**Authors:** Nia Gecheva, Stefan Tserovski, Alexander Gerchev, Milka Dikova, Pavel Georgiev, Hristo Georgiev

**Affiliations:** Department of Orthopedics and Traumatology, Medical University of Sofia, Sv. Georgi Sofiyski Blvd 1, Sofia 1431, Bulgaria; Clinics of Pediatric Orthopedics, University Hospital of Orthopedics “Prof. B. Boychev”, 56 Nikola Petkov Blvd, Sofia 1614, Bulgaria; Department of Orthopedics and Traumatology, Medical University of Sofia, Sv. Georgi Sofiyski Blvd 1, Sofia 1431, Bulgaria; Clinics of Pediatric Orthopedics, University Hospital of Orthopedics “Prof. B. Boychev”, 56 Nikola Petkov Blvd, Sofia 1614, Bulgaria; Department of Orthopedics and Traumatology, Medical University of Sofia, Sv. Georgi Sofiyski Blvd 1, Sofia 1431, Bulgaria; Clinics of Pediatric Orthopedics, University Hospital of Orthopedics “Prof. B. Boychev”, 56 Nikola Petkov Blvd, Sofia 1614, Bulgaria; Clinics of Pediatric Orthopedics, University Hospital of Orthopedics “Prof. B. Boychev”, 56 Nikola Petkov Blvd, Sofia 1614, Bulgaria; Department of Pediatrics, Medical University of Sofia, Sv. Georgi Sofiyski Blvd 1, Sofia 1431, Bulgaria; Department of Orthopedics and Traumatology, Medical University of Sofia, Sv. Georgi Sofiyski Blvd 1, Sofia 1431, Bulgaria; Clinics of Pediatric Orthopedics, University Hospital of Orthopedics “Prof. B. Boychev”, 56 Nikola Petkov Blvd, Sofia 1614, Bulgaria; Department of Orthopedics and Traumatology, Medical University of Sofia, Sv. Georgi Sofiyski Blvd 1, Sofia 1431, Bulgaria; Clinics of Pediatric Orthopedics, University Hospital of Orthopedics “Prof. B. Boychev”, 56 Nikola Petkov Blvd, Sofia 1614, Bulgaria

**Keywords:** ankle, pediatric, infection, histologic examination

## Abstract

We present a case of a 2-year-old child with persistent synovitis of the right ankle joint accompanied by a subfebrile temperature. The child had received Bacillus Calmette-Guérin vaccination and had minimal laboratory abnormalities. Thorough imaging studies revealed a well-defined osteolytic lesion of the distal tibial metaphysis with partial physeal and epiphyseal involvement. Targeted biopsy with polymerase chain reaction and histopathological examination demonstrated caseating granulomatous inflammation with Langhans-type giant cells and confirmed *Mycobacterium tuberculosis* without Rifampicin resistance. Surgical curettage was performed with preservation of the physeal circumference, followed by weight-adjusted four-drug anti-tuberculous therapy for 2 months and prolonged dual therapy, with a total treatment duration of 9 months. At 18-month follow-up, the child was asymptomatic, with full range of motion and preserved longitudinal tibial growth. Early diagnosis, targeted biopsy, appropriate surgical management, and prolonged chemotherapy are critical for optimal outcomes.

## Introduction

Bone and joint tuberculosis (TB) accounts for ~2.2%–4.7% of all TB cases in Europe and the USA and represents around 10%–15% of extrapulmonary TB cases [1, 2]. In children, osteoarticular TB is rare and may be considered exceptional in BCG-vaccinated (Bacillus Calmette-Guérin) patients with a negative Mantoux test [3, 4]. Only isolated cases of tuberculous osteitis in very young, including non-ambulatory, children have been reported, particularly involving the distal tibial metaphysis. Kao *et al*. reported physeal involvement in 19 children with long-bone TB, and Erol *et al*. documented distal femoral and proximal tibial lesions that were frequently referred to as suspected bone tumors. Similar to our case, these reports describe significant diagnostic challenges due to the atypical clinical presentation and the absence of pronounced systemic manifestations [5–8].

Chronic ankle pain or synovitis and a lytic metaphyseal lesion in early childhood have a broad differential diagnosis. Subacute bacterial or fungal osteomyelitis, *Kingella kingae* infection, Langerhans cell histiocytosis, simple or aneurysmal bone cysts, cartilage tumors, and osteoid osteoma may mimic tuberculous osteitis clinically and radiologically [2, 3, 6, 9, 10]. The diagnostic challenge is therefore to recognize an indolent course, persistent symptoms, limited response to initial treatment, and an epidemiological context in which TB remains plausible, and then to obtain tissue for histopathological, microbiological, and molecular confirmation [6, 9].

## Case report

A 2-year-old child was admitted to the Pediatric Orthopaedics Clinic. The patient was a citizen of a non-European Union country, from a family of high socioeconomic status. Immunizations were complete, including the BCG.

Since the age of 1 year and 4 months, the child had persistent synovitis of the right ankle joint accompanied by a subfebrile temperature of up to 37.7°C. Initial treatment in the country of origin included outpatient management for transient synovitis and subsequent hospitalization with a diagnosis of juvenile rheumatoid arthritis. After magnetic resonance imaging (MRI), distal tibial osteomyelitis was suspected. A partial needle biopsy and a surgical synovial biopsy demonstrated chronic granulomatous inflammation.

At admission to our clinic, the child was afebrile with normal neuropsychological and physical development. The patient was non-ambulatory due to protective avoidance of weight bearing on the right ankle. Clinical examination revealed swelling of the right ankle joint, increased local temperature, and a limited, painful ankle range of motion (S–0–0–15), while subtalar motion was preserved.

Laboratory findings showed no substantial inflammatory activity: erythrocyte sedimentation rate was 8 mm/h; and C-reactive protein was 8 mg/l. Haematological indices were: hemoglobin, 110 g/l (11.0 g/dl); hematocrit, 0.316 l/l (31.6%); and red blood cell count, 4.05 × 10^12^/l. Alkaline phosphatase was 600 U/l. The Mantoux test showed an induration of 8 mm.

**Figure 1:**
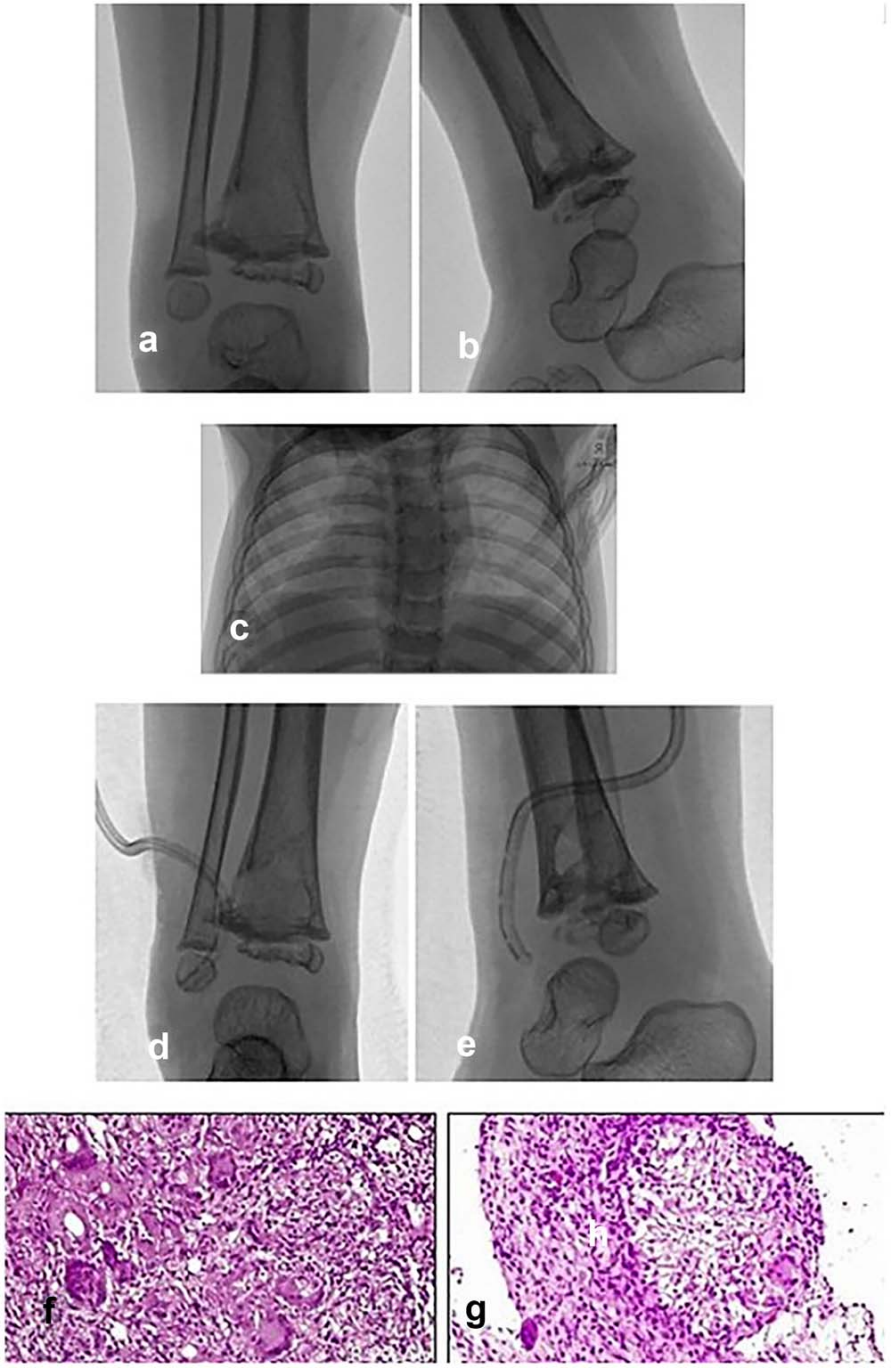
(a,b) Radiography of the right ankle revealed a well-demarcated radiolucent lesion in the distal tibial metaphysis without periosteal reaction; (c) chest radiography—normally aerated lung fields and a normal cardiac silhouette; (d,e) postoperative radiograph showing the pathologically altered metaphysis after curettage down to healthy cortical bone; (f) H&E staining demonstrating caseous necrosis surrounded by a rim of epithelioid cells, areas of tubercle formation, and scattered multinucleated Langhans-type giant cells within connective tissue exhibiting chronic nonspecific inflammatory infiltration with numerous leukocytes; (g) decalcified bone tissue showing a well-formed tubercle composed of epithelioid cells, Langhans-type multinucleated giant cells, sparse lymphocytes, and surrounding connective tissue.

Plain radiography of the right ankle revealed a well-demarcated radiolucent lesion in the distal tibial metaphysis without a periosteal reaction (Fig. 1).

**Figure 2:**
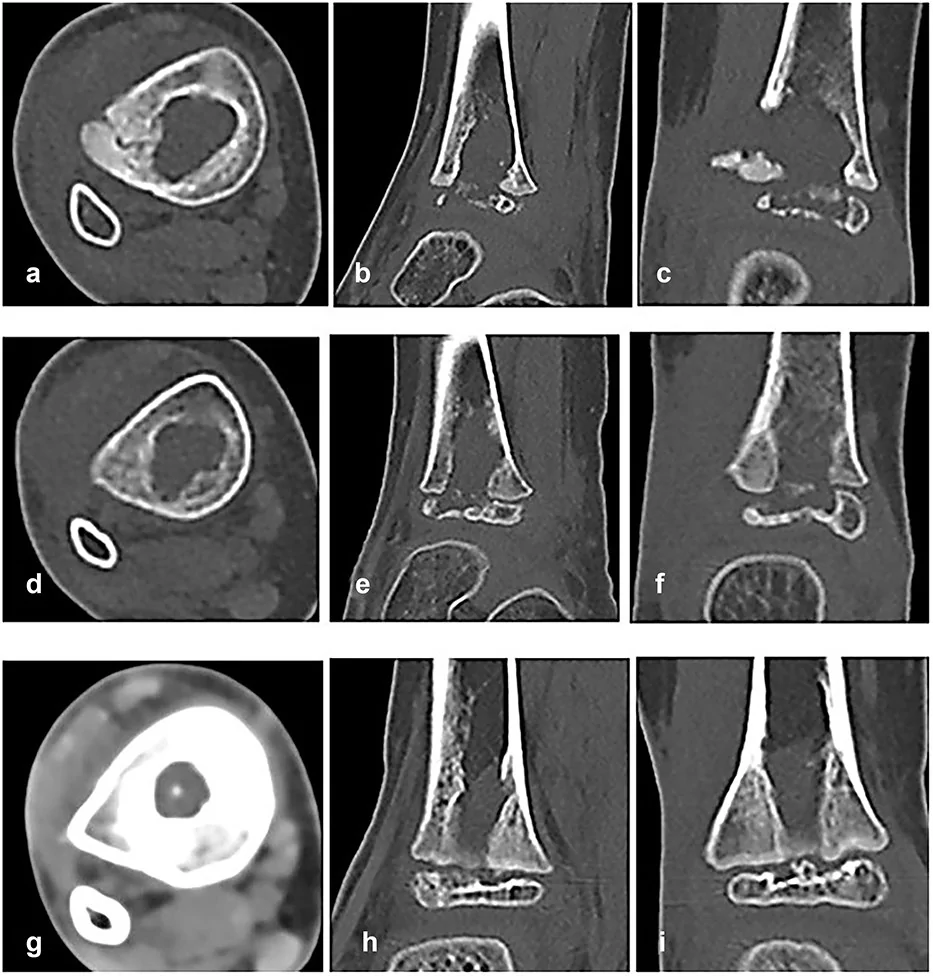
Dynamic CT evaluation in axial, sagittal, and coronal planes through the widest diameter of the lesion; (a–c) pretreatment CT scans: osteolytic metaphyseal lesion with cortical destruction along the lateral contour of the distal tibia; the lesion has well-defined margins with a focally mild osteosclerotic rim formation; maximum dimensions in the three planes are 18 × 15 mm, with a partially destroyed physis and an additional epiphyseal focus measuring 5 × 10 mm; the lesions are filled with homogeneous, structureless material of soft-tissue (parenchymal) density; (d–f) CT scans obtained 6 months after surgery showing a postoperative defect in the metaphysis and epiphysis with well-defined margins; the epiphysis appears fragmented and markedly comminuted; (g–i) CT scans at 18 months after surgery persistence of the postoperative metaphyseal defect with significantly reduced dimensions; the previously fragmented epiphyseal portions have consolidated into a unified structure; the physis is clearly delineated, with no evidence of secondary epiphysiodesis.

Computed tomography (CT) demonstrated an osteolytic lesion with lateral metaphyseal destruction and partial involvement of the distal epiphysis (Fig. 2).

MRI of the right ankle, performed prior to admission to our institution, showed an altered signal within the distal tibial osseous structure, consistent with osteomyelitis. The abnormality extended both proximally and distally to the distal tibial physis, involving the adjacent metaphysis, distal epiphysis, and intramedullary distal diaphysis. Marked tibiotalar synovitis was present, with probable synovial proliferative tissue. Assessment of the synovium and soft tissues was limited because contrast-enhanced sequences were not obtained.

Through an anterolateral approach and the removal of scar tissue from the previous biopsy, inflammatory changes were observed in the surrounding soft tissues and periosteum of the distal tibia. Partial decortication exposed a metaphyseal defect ~5 cm in longitudinal extent, centrally crossing the physis and reaching the distal tibial epiphysis. The hyaline cartilage was preserved. The cavity was filled with a soft, gelatinous, yellow-brown substance.

Careful curettage of the pathological metaphyseal and diaphyseal cancellous bone was performed up to healthy cortical bone, followed by extensive lavage with normal saline and hydrogen peroxide. Through a minimal central opening in the physis, curettage of the epiphyseal lesion was performed. The circumferential physis surrounding the defect remained intact. The wound was closed in layers with active drainage for 48 h. The early postoperative period was uneventful with primary wound healing.

Histopathological examination demonstrated caseating granulomatous inflammation with epithelioid cell granulomas. Real-time polymerase chain reaction (PCR) (Xpert MTB/RIF Ultra, assay version 4) detected *M. tb*. at low levels without rifampicin resistance. Microbiological cultures were negative.

Following surgical intervention, the patient was initiated on a 9-month anti-TB chemotherapy regimen appropriate for osteoarticular TB. The intensive phase comprised a daily four-drug combination of isoniazid (5 mg/kg/day), rifampicin (10 mg/kg/day), pyrazinamide (25 mg/kg/day), and ethambutol (15–25 mg/kg/day) administered for 2 months.

Subsequently, a continuation phase with isoniazid and rifampicin was maintained for an additional 7 months.

An ankle–foot orthosis was prescribed and maintained for 9 months to ensure limb stabilization and protection during recovery.

CT examinations at 6 and 18 months postoperatively demonstrated progressive reduction of the osseous defect, reconstitution of the metaphyseal bone structure, and restoration of the distal tibial epiphysis. At the final follow-up, the child was pain-free, ambulatory, and without angular deformity or evidence of growth disturbance (Fig. 2).

**Figure 3:**
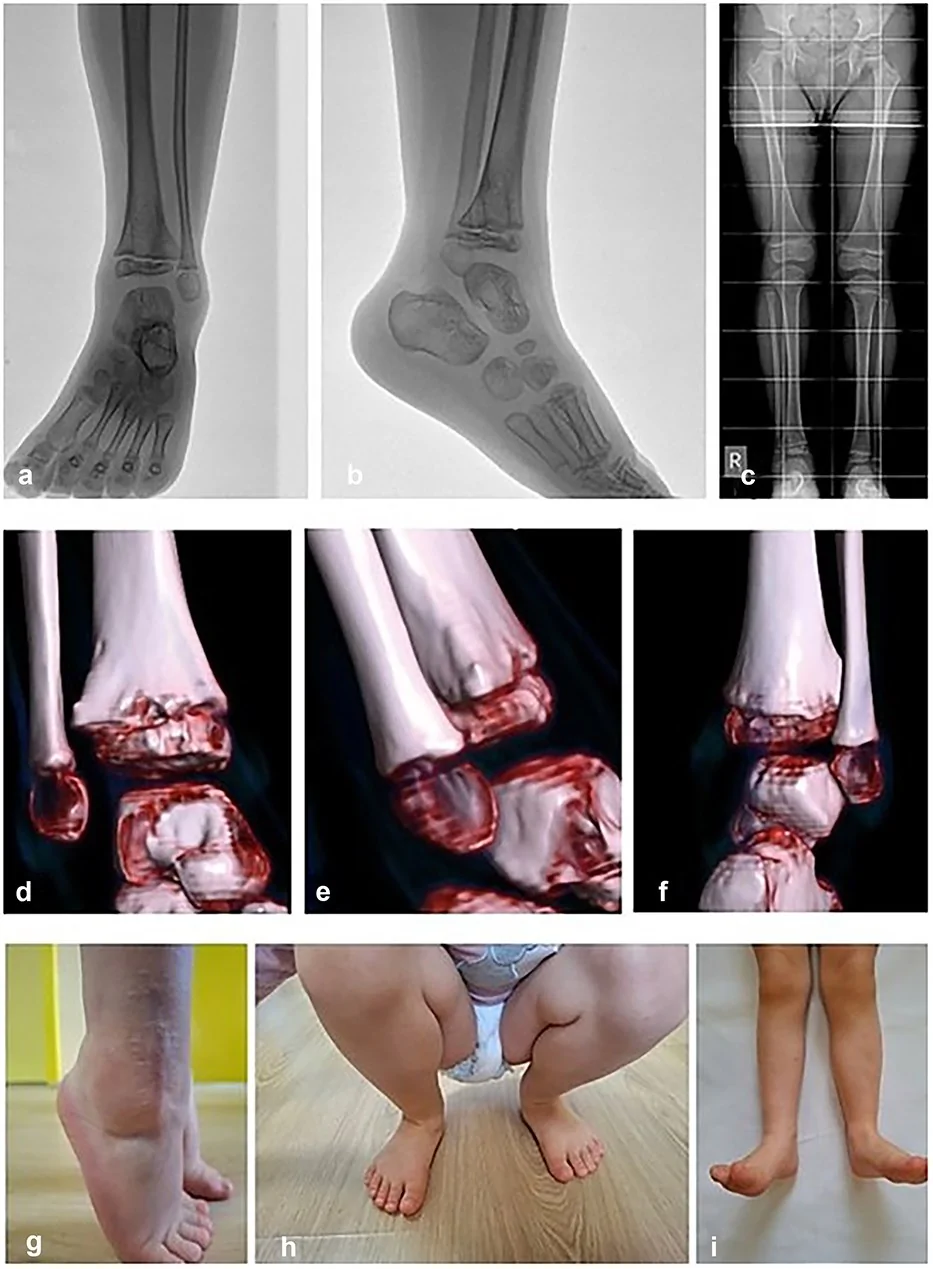
(a–c) Plain radiographs obtained 1 year and 6 months postoperatively demonstrating advanced consolidation of the lesion; the distal tibial physis shows preserved growth, and the lower limbs are of equal length; (d–f) three-dimensional CT scans of the ankle and foot 18 months after surgery; (g–i) clinical photographs demonstrating full ROM of the ankle and foot.

At 1 year and 6 months postoperatively, the child had no limitations in physical activity. The range of motion (ROM) of the ankle and foot was full, and tibial growth was preserved (Fig. 3).

## Discussion

The initial presentation with persistent ankle synovitis, avoidance of weight-bearing, nonspecific laboratory findings, and no evidence of concomitant pulmonary disease made tuberculous osteitis of the distal tibia particularly difficult to suspect. Because the predominant manifestation was monoarthritis, transient synovitis and juvenile idiopathic arthritis were also relevant early clinical considerations. Once the focal lytic lesion was recognized, the differential diagnosis broadened to include chronic pyogenic or fungal osteomyelitis, simple or aneurysmal bone cysts, cartilage tumours, osteoid osteoma, and lytic neoplasms, alongside osteoarticular TB [6, 11].

Tuberculous osteitis can overlap clinically and radiologically with chronic infection and bone tumours, especially when respiratory symptoms are absent and inflammatory markers are only minimally elevated [9, 11]. Imaging is therefore used to define the extent of disease and guide sampling rather than to establish the diagnosis in isolation. In this case, the indolent course, persistent synovitis, and metaphyseal/epiphyseal involvement justified a targeted biopsy. Caseating granulomatous inflammation together with a positive Xpert MTB/RIF Ultra testing established the diagnosis of tuberculous osteitis [6, 9, 11, 12]. In our patient, the definitive diagnosis was established through histopathological identification of granulomatous inflammation with caseation.

Surgical curettage of the lesion effectively removes pathological tissue and facilitates spontaneous restoration of the cancellous bone structure [2, 5, 7, 9]. In the present case, the minimal central transphyseal approach used to curette the epiphyseal lesion did not compromise longitudinal bone growth, as the circumferential integrity of the physis was preserved. According to Kao *et al*., even with more extensive curettage, the risk of permanent physeal damage remains minimal [8].

In our patient, the combination of appropriate anti-tuberculous therapy and preservation of the intact medial portion of the epiphysis likely allowed the ossification center to consolidate and grow symmetrically with that of the unaffected tibia. Anti-tuberculous therapy was administered according to established treatment protocols [13].

## Conclusion

In pediatric patients presenting with bone lesions in the ankle and foot region, we recommend thorough imaging evaluation with MRI or CT to accurately delineate the extent of the lesion, followed by a targeted biopsy with PCR *in vitro* diagnostic testing for *M. tb*. and histopathological examination. Complete surgical curettage of the pathological tissue, combined with appropriate anti-tuberculous therapy when TB is confirmed, offers a high likelihood of restoration of the epiphyseal complex and preservation of normal tibial growth.

## References

[1] Pigrau-Serrallach C, Rodríguez-Pardo D. Bone and joint tuberculosis. Eur Spine J 2013;22:556–66. 10.1007/s00586-012-2331-y PMC3691411 22711012

[2] Rasool MN . Osseous manifestations of tuberculosis in children. J Pediatr Orthop 2001;21:749–55. 10.1097/01241398-200111000-00009 11675548

[3] Malik S, Joshi S, Tank JS. Cystic bone tuberculosis in children: a case series. Indian J Tuberc 2009;56:220–4. 20469735

[4] Houshian S, Poulsen S, Riegels-Nielsen PR. Bone and joint tuberculosis in Denmark: increase due to immigration. Acta Orthop Scand 2000;71:312–5. 10.1080/000164700317411942 10919306

[5] Alasaad H, Diri D, Mhana SAA et al. Solitary distal tibia tuberculosis in a child, effectively treated by chemotherapy following surgery: a case report. Int J Surg Case Rep 2024;115:109289. 10.1016/j.ijscr.2024.109289 38266362 PMC10832481

[6] Gümüştaş SA, Çağırmaz T, Orak MM et al. Cystic tuberculosis osteomyelitis of the distal tibia in infancy. Turk Pediatri Ars 2017;52:53–6. 10.5152/TurkPediatriArs.2017.2479 28439203 PMC5396824

[7] Erol B, Topkar MO, Basar H et al. Solitary cystic tuberculosis of the distal femur and proximal tibia in children. J Pediatr Orthop B 2015;24:315–20. 10.1097/BPB.0000000000000193 26035351

[8] Kao HK, Yang WE, Shih HN et al. Physeal change after tuberculous osteomyelitis of the long bone in children. Chang Gung Med J 2010;33:453–60. 20804676

[9] Faroug R, Psyllakis P, Gulati A et al. Diagnosis and treatment of tuberculosis of the foot and ankle: a literature review. Foot (Edinb) 2018;37:105–12. 10.1016/j.foot.2018.07.005 30359882

[10] Ceroni D, Cherkaoui A, Combescure C et al. Differentiating osteoarticular infections caused by Kingella kingae from those due to typical pathogens in young children. Pediatr Infect Dis J 2011;30:906–9. 10.1097/INF.0b013e31821c3aee 21494171

[11] Benjelloun M, Tijani N, BenLahlou Y et al. Tibial osteitis caused by *Mycobacterium tuberculosis*. Access Microbiol 2025;7:000960.v4. 10.1099/acmi.0.000960.v4 PMC12284410 40703940

[12] Opota O, Mazza-Stalder J, Greub G et al. The rapid molecular test Xpert MTB/RIF Ultra: towards improved tuberculosis diagnosis and rifampicin resistance detection. Clin Microbiol Infect 2019;25:1370–6. 10.1016/j.cmi.2019.03.021 30928564

[13] Oliwa J, Graham S. *Diagnosis and Management of Tuberculosis in Children and Adolescents: A Desk Guide for Primary Health Care Workers (4th ed.)*. The Union, International Union Against Tuberculosis and Lung Disease (2023).

